# Neuroimmune mechanisms in Krabbe's disease

**DOI:** 10.1002/jnr.23804

**Published:** 2016-09-17

**Authors:** Gregory B. Potter, Magdalena A. Petryniak

**Affiliations:** ^1^ Denali TherapeuticsSouth San FranciscoCalifornia; ^2^ Department of PediatricsOregon Health and Science UniversityPortlandOregon

**Keywords:** globoid cell leukodystrophy, DOID:10587, twitcher, RRID:IMSR_JAX:000845, twi‐5J, RRID:IMSR_JAX:003613, microglia, NIFCELL:NIFEXT_170, GALC, PR:0000007811

## Abstract

Neuroinflammation, activation of innate immune components of the nervous system followed by an adaptive immune response, is observed in most leukodystrophies and coincides with white matter pathology, disease progression, and morbidity. Despite this, there is a major gap in our knowledge of the contribution of the immune system to disease phenotype. Inflammation in Krabbe's disease has been considered a secondary effect, resulting from cell‐autonomous oligodendroglial cell death or myelin loss resulting from psychosine accumulation. However, recent studies have shown immune activation preceding clinical symptoms and white matter pathology. Moreover, the therapeutic effect underlying hematopoietic stem cell transplantation, the only treatment for Krabbe's disease, has been demonstrated to occur via immunomodulation. This Review highlights recent advances in elaboration of the immune cascade involved in Krabbe's disease. Mechanistic insight into the inflammatory pathways participating in myelin and axon loss or preservation may lead to novel therapeutic approaches for this disorder. © 2016 The Authors. Journal of Neuroscience Research Published by Wiley Periodicals, Inc.

Leukodystrophies are the most common cause of pediatric neurodegeneration, associated with profound childhood morbidity and mortality and resulting in significant emotional and financial burden on families and society (Kohlschutter and Eichler, 2011). Although white matter degeneration is a common feature of these disorders, the activation of the CNS's innate immune response is also observed in most leukodystrophies and coincides with white matter pathology, disease progression, and morbidity (Vitner et al., 2010). Despite this, there is a major gap in our knowledge of the contribution of the immune system to disease phenotype. Krabbe's disease (KD), a leukodystrophy caused by an enzymatic defect in lysosomal galactocerebrosidase (GALC), presents in the most severe infantile form by 6 months of age, followed by death at 2 years of age (Wenger, 1997). This Review refers to neuroinflammation as inflammation characterized by reactivation of resident CNS innate immune cells (microglia) and astrogliosis, which has been previously used to describe aspects of KD pathophysiology (Snook et al., 2014; Hawkins‐Salsbury et al., 2015; Lin et al., 2015). It is important to note that there is no clear consensus on the definition or application of the term *neuroinflammation* with regard to neurodegenerative or lysosomal storage disorders. Some researchers draw a distinction between immune‐driven pathology in the brain (i.e., as seen in multiple sclerosis) and innate immune cell activation in the brain (Graeber, 2014), whereas others suggest dividing neuroinflammation between innate immune‐driven and adaptive immune‐driven neuroinflammation (Heppner et al., 2015). Nevertheless, it is clear that inflammation within the nervous system is a defining characteristic of KD. One of the earliest clinical manifestations (Heppner et al., 2015) of the KD phenotype is fever of unknown origin, which is indicative of the release of pyrogenic cytokines as part of an innate immune response. A pathologic hallmark of KD, first described by Danish neurologist Knud Krabbe, is the presence of phagocytic multinucleated (globoid) cells in the brain (Krabbe, 1916). The CNS of patients as well as that of all animal models exhibits robust astrogliosis, microglial activation, and macrophage recruitment (Wenger, 2000). Although it has been proposed that death of oligodendrocytes and the resulting demyelination trigger the neuroimmune response, recent studies that examined early pathology clearly demonstrated neuroinflammation preceding changes in or loss of myelin (Santambrogio et al., 2012; Potter et al., 2013), with reactive microglia detected in advance of reactive astrocytes (Snook et al., 2014). The trigger of inflammation is still not known; however, myelinating cells are particularly rich in GALC substrates and are thus predicted to be the primary cells responsible for initiating pathological changes in KD. This Review seeks to introduce important neuroimmune mechanisms that occur within KD. We summarize the current understanding of neuroinflammation in KD animal models and potential mechanisms that initiate inflammation and highlight interventions that modulate neuroinflammation and disease progression. By emphasizing the central role of neuroinflammation in KD, we hope to generate interest in exploring new therapies that target inflammation for the treatment of this progressive and devastating disease.

## NEUROINFLAMMATION IN ANIMAL MODELS OF KD

Disease‐causing mutations in GALC have been described for several species, including cat (Johnson, 1970; Sigurdson et al., 2002), dog (Wenger et al., 1999), monkey (Baskin et al., 1998; Wenger, 2000), sheep (Pritchard et al., 1980), and mouse (Duchen et al., 1980; Kobayashi et al., 1980; Luzi et al., 2001; Potter et al., 2013; Matthes et al., 2015). With the exception of the murine models, analysis and description of histology are performed at the end stages of the disease (Wenger, 2000). As such, critical examination of disease progression is lacking. Nevertheless, common end‐stage findings among all animal models are inflammatory markers that identify reactive microglia, astrogliosis, and accumulation of distinctive periodic acid‐Schiff (PAS)‐positive globoid cells. Globoid cells are large multinucleated cells that are often round or oval. The origin of globoid cells and their formation are under investigation. Because macrophages turn PAS positive when they phagocytose galactosylceramide, it had initially been proposed that globoid cells are infiltrating monocyte‐derived macrophages (Austin and Lehfeldt, 1965). On the other hand, microglia exposed to psychosine transform into globoid cells in vitro, whereas macrophages do not (Ijichi et al., 2013; Claycomb et al., 2014a, 2014b). Curiously, application of psychosine can cause multinucleation of U937 monocytes and HeLa, HL‐60, and HepG2 cells (Kanazawa et al., 2000), so whether endogenous accumulation of psychosine within GALC‐deficient microglia transforms only microglia into globoid cells *in vivo* remains to be determined. Globoid cells and mononuclear macrophage and microglia are most often found in weakly stained Luxol fast blue white matter tracts, indicating innate immune activation within poorly myelinated axon tracts. In addition to the white matter, PAS‐positive multinucleated globoid cells and smaller mononucleated PAS‐positive macrophages are often concentrated around blood vessels.

## KD MOUSE MODELS UNCOVER EARLY IMMUNE CELL ACTIVATION

The striking correlation between immune cell activation and accumulation in areas of demyelination observed at terminal stages of KD animal models suggests that neuroinflammation is a consequence of myelin loss or myelin debris. However, recent studies have challenged this conjecture through careful examination of murine models of KD for neuroimmune activation several weeks before overt signs of myelin loss.

**Figure 1:**
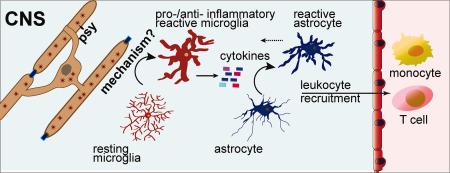
Model of innate immune response in KD. Loss of GALC enzymatic activity causes oligodendrocyte dysfunction (see also Fig. 2), which is sensed by microglia through an unknown mechanism to trigger their reactivation. Reactive microglia release cytokines and other immune signaling molecules that activate astrocytes and recruit peripheral leukocytes. Depending on the stage of the disease, reactive glia could provide either pro‐ or anti‐inflammatory effects, and the actions of innate immune signals influence disease progression.

Several mouse strains contain disease‐causing GALC mutations, including twitcher (W332X), twi‐5J (E130K), and twi^trs^ (H168C; Sakai et al., 1996; Luzi et al., 2001; Potter et al., 2013). As expected, all exhibit marked neuroinflammation at terminal stages, including microglia and astrocyte activation and macrophage infiltration. The models differ in the extent of demyelination, with the twi‐5J model showing limited CNS demyelination even at terminal stages compared with twitcher and twi^trs^. It was, in part, the finding that neuroinflammation can be robust in the absence of demyelination that led us to examine earlier aspects of immune activation in the CNS and PNS of twi‐5J mice. Indeed, we observed the presence of reactive microglia and astrocytes within the forebrain of twi‐5J mice as early as 2 weeks postnatally. Early immune activation is not restricted to twi‐5J. Remarkably, examination of twitcher hindbrain by immunohistochemistry identified ionized calcium‐binding adaptor molecule‐1^+^ reactive microglia by 2 weeks of age (Snook et al., 2014). Microglia activation was widespread by 3 weeks of age, with a significant increase in overall GFAP immunoreactivity representing astrocyte reactivation. Starting about 2 weeks of age and increasing by 3 weeks, microglia formed discrete nodules that were surrounded by hypertrophied astrocytes. By 5 weeks of age, nearly all microglia appeared amoeboid in shape, and reactive astrocytes were no longer centered around microglial nodules. These data indicate that microglia are activated first within twitcher brain, followed by astrocytes, and that eventually astrocyte reactivation is propagated beyond microglial nodules (Snook et al., 2014; Fig. 1).

Innate immune signaling might occur before histologic signs of microglia activation. Comparison of cytokine or chemokine transcript expression from total twitcher brain by qRT‐PCR revealed significant increases in *Ccl2, Il1b*, and *Tnf* at postnatal day (PND) 2 compared with controls (Santambrogio et al., 2012). TLR1 and TLR2 expression is increased by 2 weeks and 3 weeks in the hindbrain and forebrain, respectively (Snook et al., 2014). By PND 20, immune‐related genes *Ccl3, Ccl5*, and *Cxcl10* are elevated in the brain. Cytokine protein assays have demonstrated that CXCL10 and CXCL1 expression are increased compared with controls by 3 weeks postnatally, and interleukin (IL)‐6 and tumor necrosis factor (TNF)‐α are increased by 4 weeks in the hindbrain (Snook et al., 2014). Thus, cytokine and chemokine expression is elevated in presymptomatic twitcher mice and increases with disease progression.

## MECHANISMS OF NEUROINFLAMMATION

Magnetic resonance imaging studies have indicated white matter tract involvement at the time of diagnosis for infantile KD (Loes et al., 1999). Histological sections examined from a KD patient at autopsy contained active inflammation at sites of demyelination, and, because of this correlation, it was posited that oligodendrocyte death causes demyelination, which initiates inflammation by activating microglia and astrocytes, which in turn leads to further demyelination and inflammation in a feed‐forward loop. In further support of this idea, activation of immune cells followed a similar caudal‐to‐rostral gradient of demyelination, with caudal CNS tissue, such as spinal cord, exhibiting greater demyelination and inflammation compared with forebrain (LeVine et al., 1994). However, one must practice caution when predicting disease pathogenesis from examination of end‐stage diseased tissue because it is difficult to discern cause and effect from a terminal time point. Limited pathological examination of KD fetuses revealed globoid cells in both developing axonal tracts and myelinating tracts, which suggests inflammation could be occurring before any signs of demyelination or oligodendrocyte death (Martin et al., 1981). Indeed, data from studies of twitcher and twi‐5J mice have clearly shown that gliosis occurs weeks before any overt signs of oligodendrocyte dysfunction and that twi‐5J mice exhibit massive neuroinflammation without oligodendrocyte death in the forebrain. Thus, it is likely that neuroinflammation leads to oligodendrocyte dysfunction and death, which further excites innate immune pathways, leading to runaway neuroinflammation. However, what triggers neuroinflammation?

## TRIGGERS OF NEUROINFLAMMATION

GALC is ubiquitously expressed in most tissues, but most of the disease processes occur within the nervous system. Among the known GALC substrates expressed in the nervous system (GalCer, LacCer, and psychosine), the galactosphingolipid psychosine has been consistently shown to accumulate in human patients and in animal models of KD (Svennerholm et al., 1980; Whitfield et al., 2001; Esch et al., 2003; Tominaga et al., 2004). Increase in brain psychosine correlates with the appearance of cellular markers of inflammation and amplified cytokine expression (Santambrogio et al., 2012). Although high levels of psychosine can be cytotoxic to myelinating cells, its role in immune activation has not been established. It is challenging to determine the cellular concentration of psychosine *in vivo* because most measurements are performed on homogenized tissue. Measured concentrations vary widely from 10 to 1,000 pmol/mg of tissue, depending on the CNS or PNS tissue examined, with highest concentrations typically found in the sciatic nerve (White et al., 2009). The concentration of psychosine within a cell is not known. Many studies that have examined the effects of psychosine have relied on administration of exogenous psychosine to cultured cells. Typically, no effect is seen when less than 10 µM of psychosine is added, but many affects have been noted at higher concentrations, including apoptosis; disruption of sphingosine‐1‐phosphate signaling; peroxisomal and mitochondrial perturbations (Strasberg, 1986); and changes in protein kinase C (PKC), TNF, interleukin‐6, inducible nitric oxide synthase, phosphoinositide 3‐kinase, prostaglandin D2 (PGD2), and 5′‐AMP‐activated kinase expression or function (Strasberg, 1986; Ida et al., 1990; Tanaka and Webster, 1993; Giri et al., 2002, 2006, 2008; Mohri et al., 2006; White et al., 2009). In addition, exogenous psychosine can affect the function of mitochondria, reportedly by disruption of the electron transport chain and mitochondrial membrane potential (Strasberg, 1986; Haq et al., 2003) and peroxisomes (Strasberg, 1986; Haq et al., 2006). However, psychosine causes death of many cell types in culture with a threshold‐like dose–response curve, consistent with nonspecific detergent‐like effects (Suzuki, 1998). Thus, overinterpreting data generated from exogenous application of psychosine can be misleading because the cellular responses to administered psychosine likely differ from the responses to endogenous (i.e., intracellular) psychosine. Psychosine may act as a detergent within the cell, but intracellular accumulation of psychosine, such as through siRNA‐mediated knockdown of GALC within oligodendrocytes (Won et al., 2013), is a more physiological demonstration of psychosine action than exogenous administration. Although GALC is expressed by all CNS cell types, whether microglia and astrocytes accumulate psychosine at pathophysiological levels has not yet been reported. Within myelinating cells such as oligodendrocytes and Schwann cells, psychosine accumulates in lysosomes and within membrane microdomains such as lipid rafts (White et al., 2011). Thus, it is likely that lysosomal–endosomal pathways, endocytosis, and membrane receptor‐mediated signaling could be progressively disrupted as psychosine levels increase. Intracellular effects of endogenous psychosine are poorly defined. From experiments in which the effects of endogenous psychosine accumulation were observed, it has been suggested that psychosine can activate phospholipase A2, which, through generation of the bioactive lipids lysophosphatidylcholine and arachidonic acid, might activate cell death signaling cascades or the generation of reactive oxygen species (Giri et al., 2006; Won et al., 2013). In addition, changes in sphingolipid metabolism caused by lysosomal dysfunction can change the levels of sphingolipid metabolites, such as ceramide, ceramide‐1‐phosphate, and sphingosine‐1‐phosphate, which are important signaling molecules in inflammation (Maceyka and Spiegel, 2014). Additional research into cellular pathways modulated by endogenous psychosine is required to address its mechanism of action.

**Figure 2:**
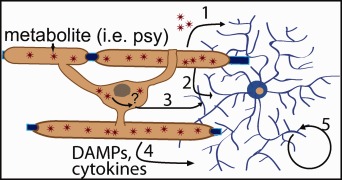
Schematic of contact‐dependent (2, 3) or secretory mechanisms (1, 4) by which oligodendrocytes could activate microglia. Secretion or exocytosis of accumulating metabolites, such as psychosine (1). Changes in membrane microdomains or membrane‐associated proteins recognized by microglia (2). Intracellular changes caused by GALC deficiency that affect membrane components, which activate microglia (3). Secretion of immune‐related molecules such as cytokines or DAMPs (4). Microglial self‐activation (5). DAMPs, danger associated molecular patterns; psy, psychosine.

Data from recent research indicate that microglia are reactive very early in the progression of mouse models of KD (Snook et al., 2014). Microglia are the innate immune cells within the CNS and are constantly sensing their environment for signs of dysfunction. They can have both proinflammatory and anti‐inflammatory actions, and the activation state of microglia is influenced by unknown mechanisms during disease progression (Saijo and Glass, 2011). A characteristic of KD is demyelination, but demyelination is a very late process in oligodendrocyte dysfunction. Earlier processes of dysfunction within GALC‐deficient oligodendrocytes could lead to activation of microglia through contact‐dependent or ‐independent mechanisms (Fig. 2).

The presence of psychosine or perturbations in lipid composition within myelin membranes because of GALC dysfunction could influence the normal expression of cell surface receptors or membrane‐associated signaling molecules (White et al., 2009, 2011). In particular, levels of membrane‐associated PKC are reduced in twitcher cells (White et al., 2009, 2011). PKC is involved in many different signaling cascades and regulates myelin gene expression and process formation within oligodendrocytes (Asotra and Macklin, 1993; Oh et al., 1997). Cell membrane perturbations could also be directly sensed by microglia, leading to their reactivation. For example, CD200 is a surface molecule expressed by oligodendrocytes that maintains microglia in a resting, inactive state (Barclay et al., 2002; Peferoen et al., 2014). Changes in CD200 localization or expression could lead to microglial reactivation. CD47, expressed within oligodendrocyte myelin, regulates the immune response by microglia. Binding of CD47 to its receptor, signal regulatory protein‐α, relays the “don't eat me” signal and prevents cells from being phagocytized by microglia (Jaiswal et al., 2009; Han et al., 2012). Although it is currently speculative, the role of surface molecules such as CD200 and CD47 and oligodendrocyte cell membrane perturbations in KD will be an informative avenue for future research.

Intracellular responses, likely influenced by GALC mutations such as endoplasmic reticulum stress, oxidative stress, metabolic disturbances, or production of misfolded proteins, can lead to oligodendrocyte stress (Peferoen et al., 2014), which could trigger an inflammatory response. For instance, age‐ and region‐dependent patterns of metabolic disturbances within oligodendrocytes correlate with microglia activation and neurodegeneration (Meisingset et al., 2013). When stressed, oligodendrocytes can release cytokines, such as CCL2, IL‐6, IL‐8, and IL‐1β, which can recruit or reactivate microglia. After reactivation, microglia becomes a major source of cytokine and chemokine expression within KD brains. Notably, all these cytokines are elevated in KD or twitcher tissue (Formichi et al., 2007; Luzi et al., 2009; Santambrogio et al., 2012). Danger‐associated molecular patterns such as ATP or TLR2 could be released by oligodendrocytes and act on microglia P2X7 or TLR receptors, respectively. In support of this idea, TLR2 is upregulated at 2 weeks of age in twitcher hindbrain, coincident with morphological evidence of microglial activation (Snook et al., 2014). Finally, it is also possible that GALC‐deficient microglia or astrocytes self‐activate, but this remains to be tested experimentally.

## MODULATION OF NEUROINFLAMMATION CHANGES KD PATHOLOGY

### Hematopoietic Stem Cell/Bone Marrow Transplantation

The only treatment currently available for KD is hematopoietic stem cell transplantation (HSCT) with bone marrow or umbilical cord blood before the onset of symptoms (Escolar et al., 2005). HSCT can prolong survival in KD, leading to improvement in nerve conduction studies in addition to transient arrest in CNS symptoms (Escolar et al., 2005; McGraw et al., 2005). Survival is prolonged for several years, but progressive neurological degeneration continues. The mechanisms by which HSCT prolongs survival are not known, but HSCT experiments in twitcher mice resulted in decreased expression of immune‐related molecules such as Cxcl10, Ccl2, Ccl3, Ccl4, and Ccl5 and delayed demyelination, which was not explained by correction of GALC deficiency (Wu et al., 2001; Siddiqi et al., 2006; Luzi et al., 2009; Santambrogio et al., 2012). Thus, HSCT likely acts to dampen neuroinflammation and thereby delay disease progression.

### Anti‐Inflammatories

Daily treatment of twitcher mice (starting from PND 10) with minocycline (a semisynthetic tetracycline that inhibits microglia activation) or indomethacin (a nonsteroidal anti‐inflammatory) resulted in downregulation of expression of *Ccl3, Ccl5, Il1a, Cxcl10*, and *Tnf* and in partial reduction of macrophages and globoid cells in brain tissues of treated twitcher mice. These changes in inflammation strongly correlated with a delayed onset of symptoms and significant, albeit modest, prolongation of life span (Luzi et al., 2009). PGD2 signaling can influence inflammation. Blockade of hematopoietic PGD synthase (HPGDS), which is responsible for the production of PGD2, in twitcher mice with an HPGDS inhibitor resulted in significant suppression of astrogliosis and demyelination and reduction in twitching and spasticity (Mohri et al., 2006). Oligodendroglial apoptosis was also reduced in twitcher mice treated with an HPGDS inhibitor. Thus, PGD2 is a neuroinflammatory molecule that amplifies the pathological response to demyelination in twitcher.

### Transgenic Mice

Twitcher/IL‐6‐deficient mice have a more severe disease than regular twitcher mice. In particular, they have an earlier onset of twitching, a greater number of PAS‐positive cells, an increased gliotic response around vessels, an elevated level of TNF‐α, and a compromised blood–brain barrier (BBB). Thus, IL‐6 deficiency causes enhanced pathology in twitcher, suggesting that IL‐6 plays a protective role in mouse models of KD (Pedchenko and LeVine, 1999).

The critical role of microglia and macrophages in ameliorating twitcher disease pathology was demonstrated by cross‐breeding *twitcher* mice with osteopetrotic (Csf1^op^, *op)* mice, which lack macrophages and have reduced microglia activation (Kondo et al., 2011). Twitcher+op mice have few microglia and macrophages in the white matter and exhibit a more severe clinical phenotype compared with twitcher mice. Twitcher+op double mutants die significantly sooner than twitcher mice, with more exacerbated neurological symptoms. The number of nonmyelinated axons in the spinal cord is significantly higher in twitcher+op mice than in twitcher mice at 45 days of age. The difference appears to be due to impaired remyelination in twitcher+op mice rather than accelerated demyelination. The levels of psychosine do not correlate with the severity of disease because psychosine levels in twitcher+op mice were lower than those in twitcher. Overall, these results indicate the beneficial actions of microglia and macrophage to counteract demyelination during twitcher disease progression.

TNF is an inflammatory cytokine that is robustly elevated in twitcher CNS and PNS. Data from mouse models of experimental encephalitis suggest that TNF exerts its actions through the TNF‐receptor 1 (TNF‐R1) in the brain. To evaluate the function of TNF signaling in the brain, twitcher/TNF‐R1‐deficient mice were generated (Pedchenko et al., 2000). Contrary to expectations, TNF‐R1 deficiency failed to alter the clinical and pathological course in twitcher, with no statistical evidence for any differences between twitcher and twitcher/TNF‐R1‐null mice for life span, weight loss, onset day of twitching, demyelination, astrocyte gliosis, and macrophage infiltration. However, when challenged with lipopolysaccharide, TNF‐R1‐deficient twitcher mice showed an exacerbated response and increased breakdown of the BBB. Recent clinical studies in patients treated with TNF antagonists have indicated that TNF has more complex immune regulatory properties than previously considered (Van Hauwermeiren et al., 2011). Animal studies have shown that TNF can exert immune‐suppressive functions and that interaction of TNF with TNF‐R2 seems to play an important role, in particular for the function of regulatory T cells and myeloid‐derived suppressor cells (Cope et al., 1997; Chen et al., 2007, 2013; Sade‐Feldman et al., 2013). Thus, it is possible that TNF signaling via non‐TNF‐R1‐mediated pathways might influence peripheral immune signaling in twitcher disease pathology, which could be tested via knockout of TNF or TNF‐R2 in twitcher mice or by the application of TNF antagonists to twitcher mice.

### Gene Therapy

Replenishment of GALC activity via viral‐mediated gene therapy is an attractive potential therapy for KD. Indeed, different forms of gene therapy in twitcher have yielded moderate, if temporary, success. Twitcher mice provided with CNS‐targeted adeno‐associated virus 2/5 (AAV2/5):GALC gene transfer showed alleviation of morphological and functional deterioration in the brain but not in the spinal cord, with reduced axonopathy and gliosis and significantly prolonged life span (Lin et al., 2011). Similarly, cerebellum‐targeted gene therapy with AAV2/5:GALC corrected enzymatic deficiency by direct transduction to Purkinje cells and cross‐correction in other cell types in the cerebellum, leading to the amelioration of both neuroinflammation and demyelination (Lin et al., 2015). Likewise, CNS‐targeted lentiviral‐mediated transfer of GALC in neonatal twitcher mice resulted in transitory reduction of psychosine levels and inflammation and delay in pathology (Lattanzi et al., 2010). Administration of AAVrh10:GALC viral particles via intracerebroventricular, intracerebellar, and intravenous injection in neonatal twitcher mice resulted in GALC activity in CNS, PNS, and some peripheral organs (Rafi et al., 2012). In correlation with a significantly improved life span and preserved myelination, reactive astrocytes and microglia were dramatically reduced in treated twitcher mice. Altogether, these experiments have demonstrated that prolonged life span and reduced pathology mediated by gene therapy are invariably correlated with reduced inflammation. Notably, combination of bone marrow transplantation with gene therapy prolongs life span even better than each treatment alone, indicating that replacement of GALC enzymatic activity is most effective when accompanied by modulation of immunity (Rafi et al., 2015).

## FUTURE PROSPECTS

Similarly to most leukodystrophies, neuroinflammation in KD was considered a late effect, resulting from oligodendrocyte death or myelin loss. However, recent research with two different mouse models of KD have demonstrated significant astrocyte and microglia reactivation and cytokine elevations *in advance of demyelination or oligodendrocyte loss* (Santambrogio et al., 2012; Potter et al., 2013). It is increasingly clear that neuroinflammation, triggered by GALC dysfunction, is an early event in animal models of KD pathogenesis. Understanding the cellular mechanisms that trigger inflammation the primary cells that initiate and respond to the inflammatory stimuli and identifying key immune signaling pathways involved in disease progression are critical areas for future research.

## CONFLICT OF INTEREST STATEMENT

G.B.P. is an employee of Denali Therapeutics and has no competing financial interests. M.A.P. has no competing interests.

## ROLE OF AUTHORS

GBP prepared the figures. GBP and MAP wrote the Review.
